# Investigating the genetic basis of salt-tolerance in common bean: a genome-wide association study at the early vegetative stage

**DOI:** 10.1038/s41598-024-55403-z

**Published:** 2024-03-04

**Authors:** Lorenzo Raggi, Leonardo Caproni, Simona Ciancaleoni, Roberto D’Amato, Daniela Businelli, Valeria Negri

**Affiliations:** 1 Dipartimento di Scienze Agrarie Alimentari e Ambientali (DSA3), Università degli Studi di Perugia, Perugia, Italy; 2 Present Address: Center of Plant Sciences, Scuola Superiore Sant’Anna, Pisa, Italy

**Keywords:** Agricultural genetics, Genetic association study, Plant breeding, Quantitative trait

## Abstract

Salinity poses a significant challenge to global crop productivity, affecting approximately 20% of cultivated and 33% of irrigated farmland, and this issue is on the rise. Negative impact of salinity on plant development and metabolism leads to physiological and morphological alterations mainly due to high ion concentration in tissues and the reduced water and nutrients uptake. Common bean (*Phaseolus vulgaris* L.), a staple food crop accounting for a substantial portion of consumed grain legumes worldwide, is highly susceptible to salt stress resulting in noticeable reduction in dry matter gain in roots and shoots even at low salt concentrations. In this study we screened a common bean panel of diversity encompassing 192 homozygous genotypes for salt tolerance at seedling stage. Phenotypic data were leveraged to identify genomic regions involved in salt stress tolerance in the species through GWAS. We detected seven significant associations between shoot dry weight and SNP markers. The candidate genes, in linkage with the regions associated to salt tolerance or harbouring the detected SNP, showed strong homology with genes known to be involved in salt tolerance in Arabidopsis. Our findings provide valuable insights onto the genetic control of salt tolerance in common bean and represent a first contribution to address the challenge of salinity-induced yield losses in this species and poses the ground to eventually breed salt tolerant common bean varieties.

## Introduction

In agriculture, stress refers to any biotic or abiotic pressure that can limit crop development and production^1^; salinity stress occurs as consequence of accumulation of ions in the soil. Saline soils are the result of natural or human-induced salinity that is often the consequence of inadequate agricultural practices such as the excessive use of fertilizers or of saline irrigation water^2^. This condition is prevalent in several Asian and Mediterranean-European countries, including Spain, Italy, Greece, Portugal and France among others^3,4^. Salinity is a significant issue that affects crop productivity worldwide. Approximately 20% of the cultivated and 33% of irrigated farmland has been classified as affected by high salt concentrations^5^ with an increasing trend^6^. Globally, the decline in cultivable soils is estimated to be between 0.3 and 1.5 million hectares per year while the decrease in soil productivity caused by salinity and sodicity (a form of salinity where the accumulation is mainly due to salts containing sodium) affects an estimated 20 to 46 million hectares^3^.

Salinity exerts negative effects on various plant development and metabolism aspects leading to physio-morphological alterations^1^. The main causes of these alterations can be attributed to (i) osmotic stress reduced water potential in the root zone, (ii) phytotoxicity effect generated by a high concentration of ions in the tissues and (iii) disproportion of nutrient uptake and/or shoot transport^7^. Salinity-induced reductions in plant growth^8^, chlorophyll content^9^ and of root and shoot biomass^10^ have consequently been reported. Identifying genes responsible for salt tolerance, understanding their function and unravelling the associated metabolic processes are crucial steps to foster a sustainable and resilient agriculture. Plants, employ different mechanisms to cope with salt stress, including osmotic balance adjustment, salt exclusion, sequestration, oxidative protection, regulation of potassium exchange, and growth regulation^11^. Considering the complexity of the involved processes, tolerance to salt is the result of the regulation of many genes^12^. Indeed, different plant gene families have been shown to be involved in salinity stress in plants with most of the evidence accumulated so far coming from Arabidopsis^13^. The maintenance of ion homeostasis is regulated by several membrane proteins responsible for ion transport. Transport-facilitating cytosolic proteins involved in intra-cellular communication are also relevant. After protein synthesis, post-translational modifications are involved in correct folding, cellular localization, protein half-life, interaction with other proteins, cell signalling and fine-tuning of protein functioning^14^. 

Common bean (*Phaseolus vulgaris* L.), a member of the legume family, holds significant importance as widely cultivated pulse, accounting for about half of the world's grain legume production and consumption for human nutrition^15^. However, as a glycophyte, this species is highly susceptible to salt in the soil which can result in marked reductions in dry matter gain in roots and shoots along with oxidative stress^16^. Indeed, these detrimental effects on growth and yield can be observed even in soils with a relatively low electrical conductivity^17^. It is also known that in common bean, salt stress affects the expression of genes involved in a variety of cellular processes including protein folding, transport of proteins across membranes, stabilization of membranes and prevention of protein inactivation^18^.

As crop species, common bean holds remarkable genetic diversity, that can potentially be reflected into traits of agronomic importance, including salt tolerance. It is known that, in this species, the effect of salt stress is genotype-dependent, with some cultivars displaying greater tolerance than others^10,19^. To date, some studies have explored the variability of bean cultivars exposed to salinity at different growth stages but mainly on a rather limited number of genotypes; the studies have been conducted at germination^10,20–23^, seedling stage^21,23–25^ and early vegetative growth^24^. In these studies, germination percentage, rates, speed indexes (at germination), plant vigour, plant survival (at seedling stage) as well as other direct measurements like root length, fresh and dry weight, were used as criteria for the estimation of plant salinity tolerance. However, screening diversity collections for salt tolerance under field conditions poses significant challenges due to stress heterogeneity, presence of confounding factors (i.e. other soil-related stresses), and/or influence of weather-related factors (e.g. temperature and relative humidity); conducting Genome-Wide Association Study (GWAS) in these conditions may result in low cost-effectiveness. In contrast, hydroponic screening offers fast, easy-to-control method that meets the high-throughput and accuracy requirements needed for GWAS^26^. It has been suggested that in hydroponic, the imposed stress may not be sufficiently gradual or could be excessively severe. Therefore, establishing optimal condition for effective discriminations between tolerant and susceptible genotypes, is a highly recommendable practice.

Increasing access to several high-throughput genotyping technologies has allowed for many studies using association mapping approaches. Several studies have reported the use of SNP markers to perform GWASs in common bean populations including genotypes of Andean and/or Mesoamerican origin. In this species some GWAS have also been performed to detect the genetic determinants involved in stress responses; in most cases drought tolerance was the object of the investigations while none considered tolerance to high salt concentrations. Andean and Mesoamerican diversity panels were developed and used to map production traits in both heat and drought stress environments^27^. Dramadri et al.^28^ evaluated a panel of 256 Andean genotypes under drought stress/non-stress conditions, among the others, candidates were proposed involved in signalling, protein modification and abscisic acid (ABA) signaling pathway. A GWAS from Hoyos-Villegas et al.^29^ explored the genetic basis of variation for drought tolerance in a Middle American diversity panel under irrigated and rainfed conditions reporting, among the others, different markers for shoot biomass under irrigation rainfed on chromosomes 2, 8 and 11. Several QTLs, within or near candidate genes playing significant role in productivity under drought stress, have been also recently proposed by Valdisser and colleagues^30^.

The objective of this study was to evaluate salinity tolerance of different common bean genotypes at both germination and seedling and under different salt concentrations; once optimal conditions were set-up, GWAS was employed to identify the genetic determinants involved in early occurring salt stress tolerance. To the purpose, the common bean diversity panel developed by the Department of Agricultural, Food and Environmental Science of the University of Perugia was used which was made accessible to the research group. The panel encompasses 192 common bean homozygous genotypes obtained mainly from landraces (179) and cultivars (13) through five successive generations of single seed descent (SSD) in isolation. The original accessions, from which the homozygous genotypes were developed, are mainly of European origin (153) while the others of American origin (39). Information of the biological materials composing the panel are available in the National Center for Biotechnology Information (NCBI) BioSample database from ID SAMN12035168 to SAMN12035359. The panel has been genotyped using a double digest Restriction-site Associated DNA sequencing (ddRAD-seq) approach that generated a dataset of 106,072 polymorphic loci. After quality control, no genotype was excluded and a dataset of 49,518 SNPs markers evenly distributed over the 11 common bean chromosomes was retained for association analyses. Raw DNA ddRAD sequencing reads are available at the European Nucleotide archive under the ID PRJEB33063 (https://www.ebi.ac.uk/ena/data/view/). The diversity panel has been already successfully used to perform GWAS and in particular to detect genetic determinants involved in the control of flowering time^31^ and of iron and zinc accumulation in seeds^31^.

## Results

### Setting up salt stress experimental conditions

**Figure 1:**
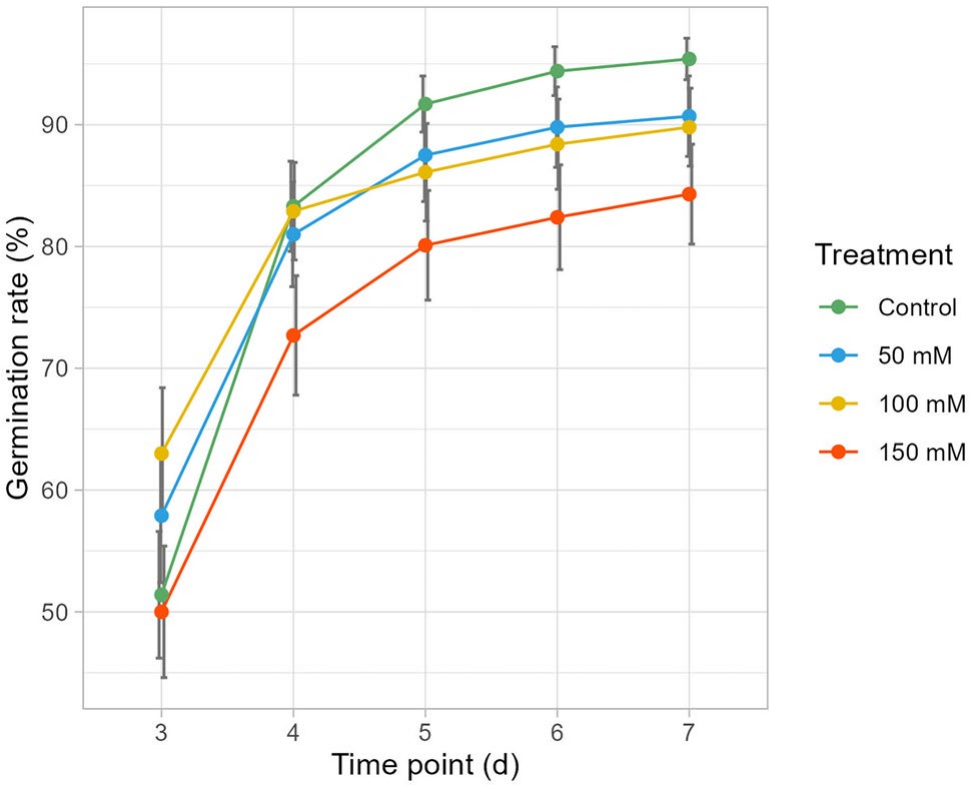
Dynamic of the germination process. Curves are percentages of the mean number of germinated seed, colours are according to the figure legend; for each day and treatment, standard error is also reported.

Tested salt concentrations reduced the germination rate of common bean lines in the initial experiment. The average percentage of germinated seeds was ≥ 50% three days after the initiation of the germination test. Noticeable differences between control and salt stress conditions became apparent from day 4 (Fig. 1). After seven days average germination percentages were 95%, 91%, 90% and 84% under Control (NaCl 0) 50, 100 and 150 mM, respectively. This clearly shows a direct reduction of germination ability as salt concentration increases (Fig. 1). More detailed results of the germination test, including the number of seeds germinated per day, genotype, treatment, and replication can be found in Table S1 (Supplementary Materials).

The Germination Rate (GR) remained relatively high throughout the test, indicating that a large proportion of the seeds had germinated by the end of the experimentation (G7) regardless of the genotype or treatment. However, the highest salt concentration (NaCl 150 mM) significantly hindered the GR compared to the control (*p* ≤ 0.05, Student’s t-test). Although not statistically significant, the Germination Potential (GP), which is influenced by the number of readily germinating seeds, and the Germination Index (GI), influenced by temporal progression of germination process, appeared to be also negative affected by salt at the different concentrations (data not shown).

When the seedlings were transferred to hydroponic conditions, plants growth was severely hindered at the two tested salt stress conditions (i.e. NaCl 100 and 150 mM); when non-stressed (i.e. control) and stressed plants were compared for shoot and root characteristics (i.e. length, fresh and dry weight), pairwise differences were always significant (Table 1). Shoot length (SL) and root length (RL) were the most affected traits with an average reduction of 71.6% and 83.3% and 63.7% and 84.7% at NaCl 100 and 150 mM, respectively.

**Table 1:** Statistics and difference analysis of seedlings traits recorded on shoot (top) and root (bottom) under control (0 mM NaCl) and the two tested salt stress conditions (100 and 150 mM NaCl): length (L), fresh weight (FW) and dry weight (DW).

Shoot	SL (cm)			SFW (g)			SDW (g)		
	NaCl	NaCl	NaCl	NaCl	NaCl	NaCl	NaCl	NaCl	NaCl
	0 (control)	100	150	0 (control)	100	150	0 (control)	100	150
Min	1.82	2.70	1.80	2.25	0.36	0.34	0.18	0.06	0.01
Max	40.70	11.00	8.60	7.82	2.50	1.67	1.51	0.42	0.68
Mean	24.19	6.88	4.03	4.97	1.47	0.99	0.47	0.23	0.27
SD	9.854	2.270	1.752	1.579	0.570	0.373	0.226	0.077	0.140
CV(%)	40.74	33.01	43.50	31.80	38.74	37.71	48.49	33.55	50.92
t-test	–	10.27***	12.09***	–	12.49***	14.71***	–	5.97***	4.35***

Root	RL (cm)			RFW (g)			RDW (g)		
	NaCl	NaCl	NaCl	NaCl	NaCl	NaCl	NaCl	NaCl	NaCl
	0 (control)	100	150	0 (control)	100	150	0 (control)	100	150
Min	13.60	2.50	1.10	0.74	0.09	0.06	0.04	0.00	0.00
Max	31.00	15.40	9.80	2.62	1.28	0.65	0.27	0.49	0.08
Mean	22.44	8.53	4.22	1.52	0.55	0.23	0.13	0.05	0.02
SD	4.142	3.230	1.686	0.516	0.326	0.131	0.055	0.079	0.018
CV(%)	18.46	37.89	39.95	33.93	59.20	56.36	43.26	147.30	86.30
t-test	–	15.89***	24.44***	–	9.53***	14.51***	–	4.53***	11.07***

Critical Student’s t-test: *n.s.* not significant, *significant at *p* ≤ 0.05, **p ≤ 0.01, ***p ≤ 0.001.

According to the ANOVA results, shoot dry weight is significantly affected by the factors “Entry” (E) and “Treatment” (T) (Table S2 A, Supplementary Materials); their interaction (i.e. ExT) was also significant showing different response of different genotypes to the same salt stress treatment. Root dry weight was also significantly affected by “E” and “T” while their interaction was not significant indicating a more constant response of the different genotypes to the different treatment levels (Table S2 B, Supplementary Materials). As expected, also the other recorded traits were affected by the factors “E” and “T” (data not shown). Indeed, the recorded phenotypes were highly correlated: RDW was positively correlated with both RL (0.84; *p* ≤ 0.001) and RFW (0.85; *p* ≤ 0.001) while SDW with SL (0.52; *p* ≤ 0.001) and SFW (0.62; *p* ≤ 0.001) (Figure S1, Supplementary Materials).

**Figure 2:**
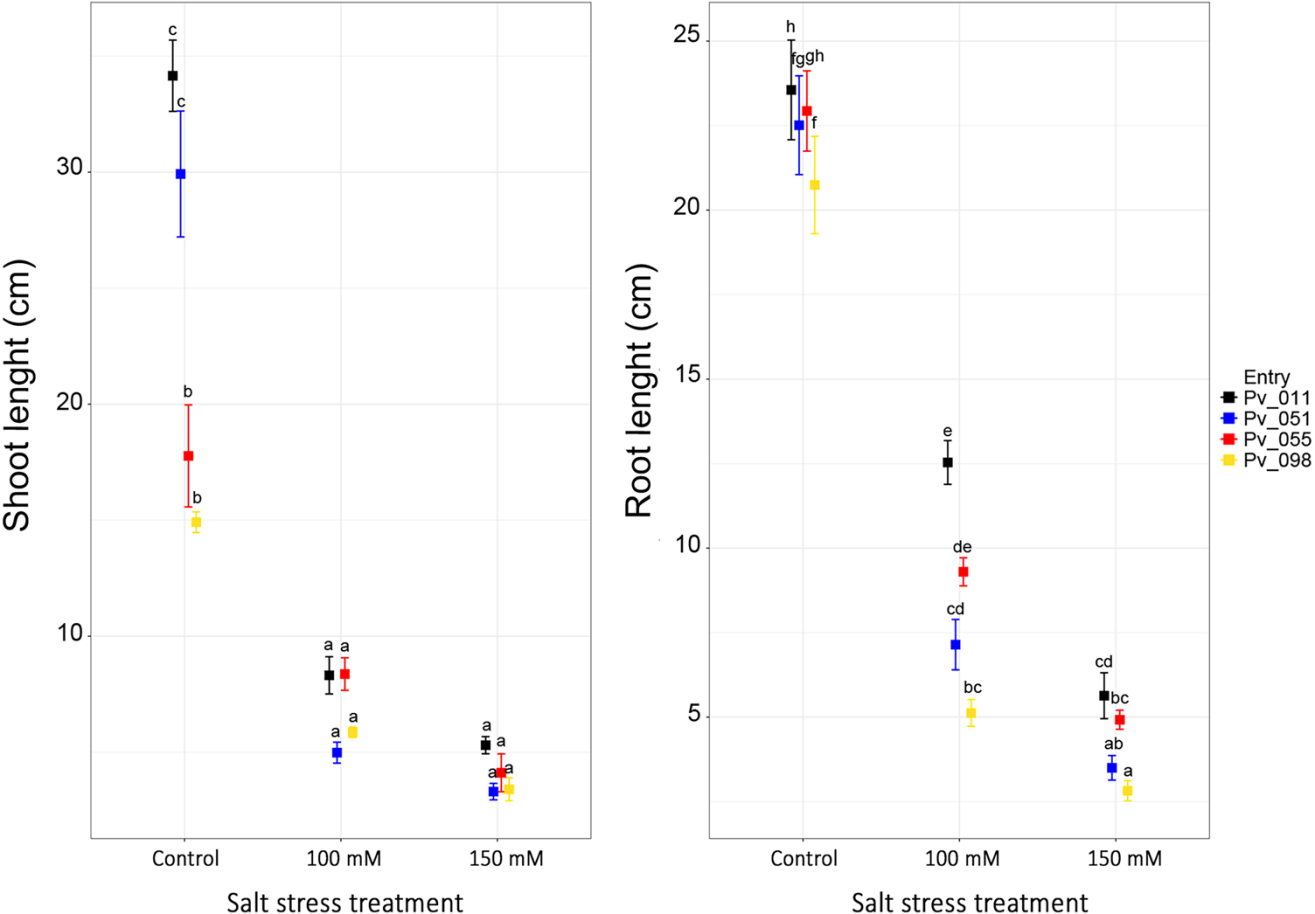
Shoot (left) and root (right) length (cm) of seedlings recorded after 10 days under hydroponic growth at NaCl 0 (control), 100 and 150 mM. Boxes indicate means; the different colours identify the different tested genotypes according to the legend; for each treatment and tested genotype, standard error is also reported.

A graphical representation of salt stress effect on shoot and root length of the different genotypes used to set up the experimental conditions of hydroponic growth (i.e. Pv_011, Pv_051, Pv_055 and Pv_098) is reported in Fig. 2. The Figure shows clearly how in control conditions, shoot length is quite different for the different tested lines and the salt treatments flatten these differences. On the other hand, under control condition root length is quite similar among the 4 tested lines, while salt treatment exerts a differentiation in length among the lines.

### Characterising the common bean collection for salt tolerance at seedling

Considering that both tested NaCl concentrations had significant detrimental effect on seedling growth, regardless of the considered genotype or traits, a concentration of NaCl 75 mM was identified as optimal for inducing salt stress and used for characterising the whole diversity panel. Indeed, the concentration was chosen to ensure the occurrence of evident salt stress effects without causing excessive damage to the seedlings. In addition, being the traits highly correlated, shoot and root dry weight (SDW and RDW, respectively) were only measured.

At the established optimal experimental conditions, a total of 130 common bean genotypes were successfully characterised for salt tolerance by recording SDW and RDW of the plants grown under both control and salt stress conditions (NaCl 75 mM). High level of variation was observed for these two traits in both conditions. Under control condition, SDW genotype mean values ranged from 0.033 g to 0.769 g, mean 0.290 g [0.202 g (Quartile 1), 0.369 g (Quartile 3)] while it ranged from 0.003 g to 0.409 g, mean 0.184 g [0.128 g (Q1), 0.231 g (Q3)] under salt stress, respectively. Under the same conditions RDW genotype mean values ranged from 0.013 g to 0.167 g, mean 0.065 g [0.045 g (Q1) 0.079 g (Q3)] while it ranged from 0.001 g to 0.106 g, mean 0.048 g [0.037 g (Q1), 0.061 g (Q3)] under salt stress, respectively. Both SDW and RDW were statistically different between control and stress conditions on average (Student’s t-test 7.86 and 5.51; p-value 1.03E−13 and 8.70E−8, respectively) with the former more affected than the latter.

#### Effect of the main factor structuring common bean diversity on shoot and root dry weight

**Figure 3:**
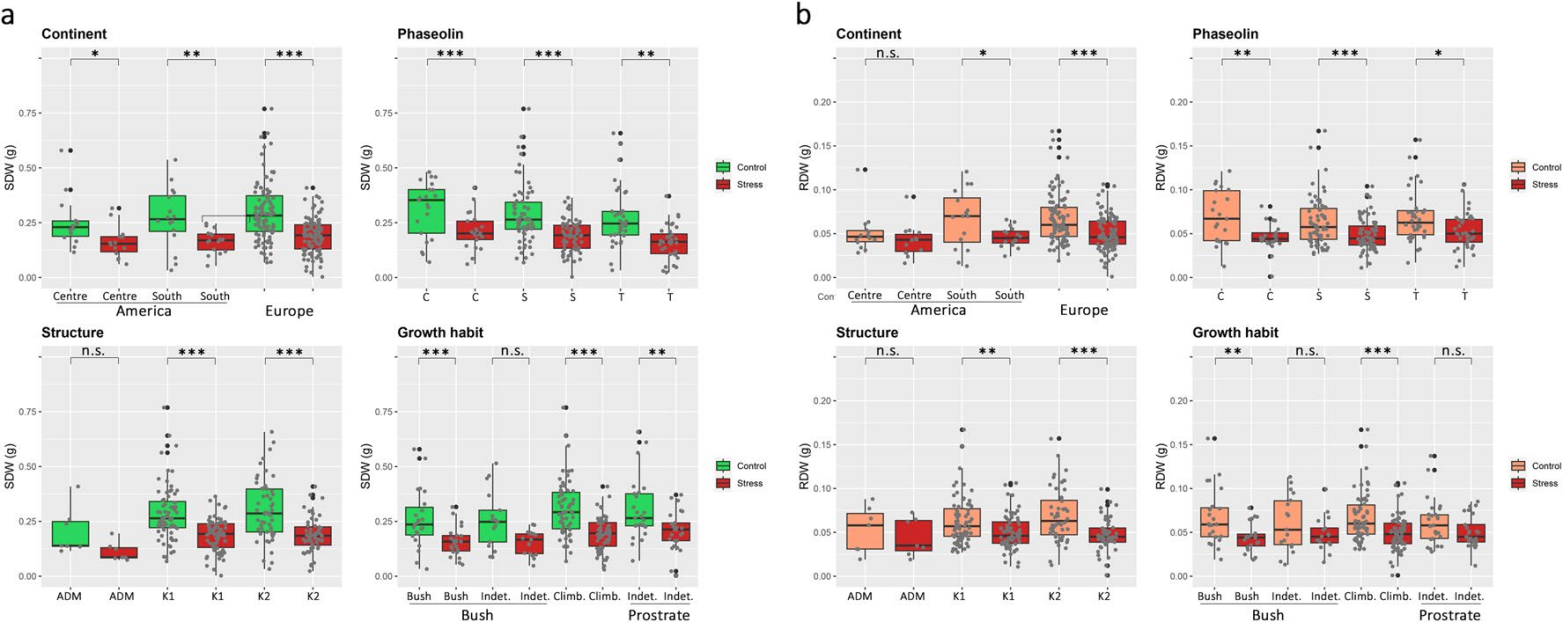
Scatterplot and boxplots of shoot dry weight (**a**) and root dry weight (**b**); data are means of 3 biological replicates of the 130 common bean genotypes grown in hydroponic culture in “Control” (NaCl 0 mM) and “Stress” (NaCl 75 mM) conditions. Data are grouped according to continent of origin (Continent), phaseoline type of the seed (Phaseolin), genetic group (Structure) and type of growth of the plant (Growth habit) of the original landraces from which each pure line has been developed^32^. The classification of each genotype for each factor was retrieved from Caproni et. al. (2019). For each level of each grouping factor considered, the significance of pairwise differences between control and stress samples is reported according to Student’s t-test: not significant (n.s.), significant at *p* ≤ 0.05 (*), *p* ≤ 0.01 (**) and *p* ≤ 0.001 (***). As for the growth habit groups description, Indet. is the abbreviation of indeterminate while Climb. of climbing.

When the genotypes were categorized based on factors describing the structure of the common bean diversity panel (i.e. geographical origin, phaseolin type, inferred genetic cluster and growth habit) and group averages of control and treated samples were compared, different results were obtained (Fig. 3). None of the three groups produced according to seed phaseoline type (C, T and S) showed any particular tolerance to salt stress; indeed, mean differences between control and treated samples of the considered groups were always significant (Fig. 3a, b, phaseoline). Similarly, grouping genotypes by continent of origin (America South, America Centre and Europe) did not affect mean salt stress tolerance of the groups with the only exception of RDW in Central America (Fig. 3a, b, continent). As for genetic origin (i.e. K1 corresponding to Mesoamerican, K2 to Andean and ADM to admixed (*q* values ≤ 0.8))^32^, Admixed genotypes showed a certain tolerance to salt stress being both SDW and RDW differences between control and treated samples not significant on average (Fig. 3a, b, structure). Finally, when growth habit is considered, differences between control and treated samples were not significant for bush-indeterminate genotypes (SDW and RDW) and prostrate indeterminate genotypes (RDW), respectively (Fig. 3a, b, growth habit).

#### Genetic diversity and salt tolerance level

**Figure 4:**
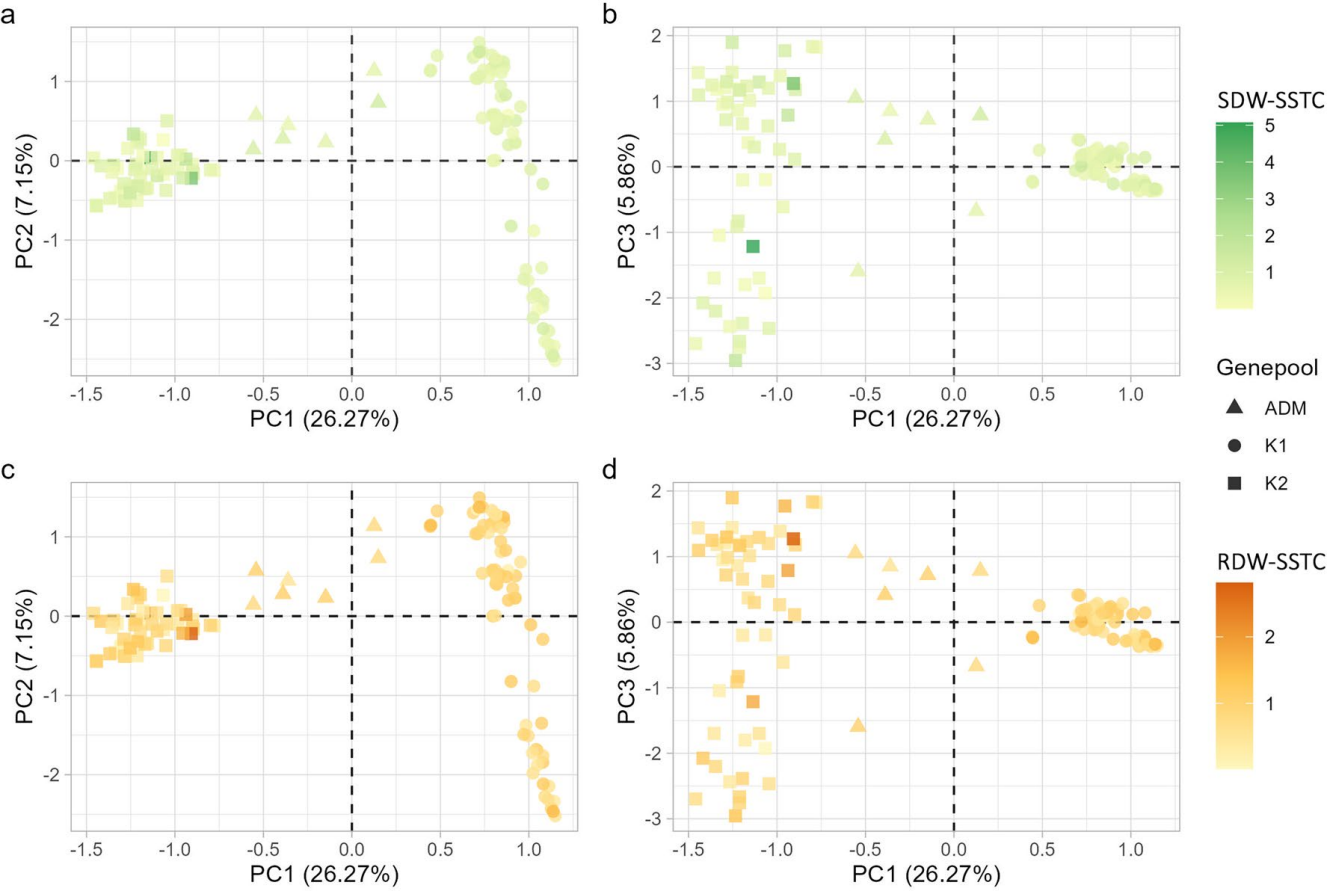
Principal component analysis based on genome-wide SNPs in approximate Linkage Equilibrium (n = 2,518); PC1 and PC2 (**a,c**) and PC1 and PC3 (**b,d**). For each genotype the symbol is according to Genepool, as described for this collection in^31,32^, while the colour to salt tolerance expressed as ‘shoot dry weight-salt stress tolerance coefficient’ (SDW-SSTC) (green, top) and ‘root dry weight-salt stress tolerance coefficient’ (RDW-SSTC) (orange, bottom). Symbols and colours are as described in the legend.

The genetic PCA showed that genepool membership is the primary factor influencing the structure of the collection. Indeed, germplasm groups defined by STRUCTURE (Figure S2, Supplementary Materials)^31,32^, correspond to distinct groups in the PCA space (Fig. 4). PC1, explaining 26.27% of the total variance, separates Andean genotypes (K2, Fig. 4) from those of Mesoamerican origin (K1, Fig. 4); genotypes of admixed ancestry lay in between the two groups (ADM, Fig. 4). PC2, explaining 7.15% of the total variance, shows the diversity among genotypes of Mesoamerican origin that are scattered along the axis (Fig. 4a, c). PC3 explains 5.86% of the total variance and depicts the diversity within the Andean group (Fig. 4b, d). Different salt tolerance level characterises the genotypes in the diversity panel considering ‘both shoot dry weight-salt stress tolerance coefficien't (SDW-SSTC) (Fig. 4, top) and r'oot dry weight-salt tolerance coefficient’ (RDW-SSTC) (Fig. 4, bottom).

### GWAS

**Figure 5:**
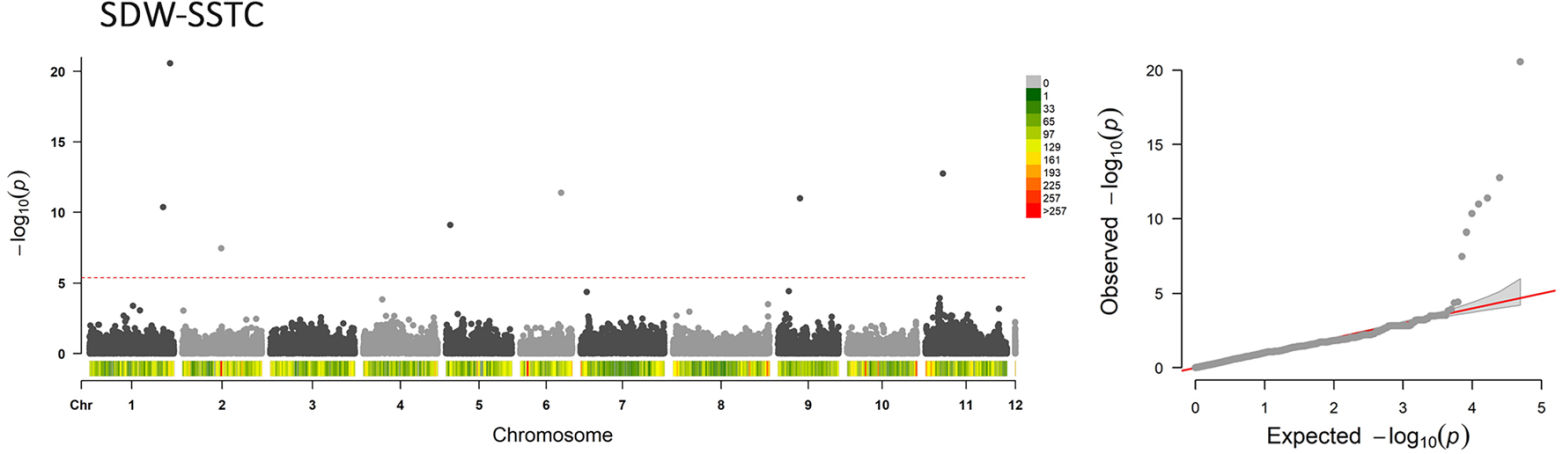
Manhattan and QQ-plot of SDW-SSTC. In the Manhattan plot, the horizontal red line corresponds to the genome-wide significance thresholds: 5.4 (Bonferroni correction based on α = 0.01). SNPs with a p-value above the selected thresholds are significantly associated with the considered trait. For each chromosome, SNP density within 1 Mb window is reported; SNP density is according to the color key. QQ-plot: scatterplot of expected (x) and observed (y) −log10 of association p values.

Heritability was relatively high for both SDW and RDW under control and stress conditions with estimated *He*_*B*_^2^ values of 0.52 and 0.67 for shoot and 0.56 and 0.62 for root under the two tested conditions, respectively. The GWAS was used to identify genomic regions involved in the mechanisms of salt stress tolerance of common bean seedlings. For the tested phenotypes, the use of 10 PC corresponded to optimal model fit (Fig. S3, Supplementary Materials). The Bonferroni correction calculated considering the number of independent recombination blocks (2443) resulted in a threshold equal to 5.4 (–log10(p)). At the considered thresholds, no significant associations were detected for RDW-SSTC; however, the analysis allowed the identification of seven significant signals of association between SNP positions and SDW-SSTC (Fig. 5).

The complete list of SNPs associated with salt stress tolerance at seedling stage, with details on chromosome position and SNP effect, is available in Table 2.

**Table 2:** List of the significant SNPs associated with shoot dry weight (SDW) under salt stress conditions identified in this study.

SNP	CH	POS	REF/ALT	MAF	Effect	SE	*p*-value^a^	Candidate
16218_44	1	44736380	T/A	0.27	−0.2031	0.03216	4.41E−11	*Pv5-593.01G186800*
17835_45	1	49131404	T/C	0.46	−0.7432	0.07049	2.76E−21	*Pv5-593.01G236100*
27203_199	2	–	T/C	0.32	−0.1643	0.03304	3.34E−08	*–*
70878_186	5	2688353	A/C	0.43	−0.2076	0.03505	7.96E−10	*Pv5-593.05G028500*
93085_71	6	17316822^b^	G/A	0.1	0.4446	0.06382	4.18E−12	*–*
139379_50	9	14050364	C/T	0.15	0.3914	0.05776	1.04E−11	*Pv5-593.09G089900*
166314_95	11	10594412	C/A	0.07	0.5662	0.07608	1.77E−13	*Pv5-593.11G099400*

For each SNP name (SNP), chromosome (CH), physical position (POS), reference and alternative allele (REF/ALT), minor allele frequency (MAF), effect (effect), standard error (SE), significance of detected association (*p*-value) and best candidate gene identified are reported.

^a^FarmCPU.

^b^60 kb window.

When aligned to the *P. vulgaris* reference genome using the BLAST tool, the DNA fragment containing SNP 70878_186 is sited within the gene *Pv5-593.05G028500* (E-value 1.92e−95, identity 99%) on chromosome 5 coding for a F-box protein 7 (FBXO7). The fragment carrying SNP 139379_50 is physically located withing the *Pv5-593.09G089900* gene (E-value 9.95e-103, identity 93%), on chromosome 9, coding for a protein phosphatase 1G. The fragment holding SNP 166314_95 is placed within a gene, *Pv5-593.11G099400* (E-value 2.95e−133, identity 96%) on chromosome 11, coding for a PROTEIN FAF-LIKE, CHLOROPLASTIC-RELATED protein.

According to BLAST results, the fragment carrying SNP 16218_44 can be attributed to a single region located in common bean chromosome 1 (E-value 2.64e−59, identity 100%) at less than 10 kb from the gene *Pv5-593.01G186800*, our best candidate for it, encoding for a ATP-BINDING CASSETTE TRANSPORTER. Also the fragment carrying SNP 17835_45 can be univocally attributed to a single region of chromosome 1 (E-value 2.21e−53, identity 99%) placed at less than 1 kb from *Pv5-593.01G236100* encoding for a RAS-RELATED PROTEIN RABA2B. The list of the most significant ortho/homologues genes in Arabidopsis of the five proposed candidates is reported in Table 3.

The fragment carrying SNP 93085_71 can be attributed with high confidence to three different regions enclosed in a 60 kb window on chromosome 6. Placed at 35 kb from one of the hits, *Pv5-593.06G070400* codes for a SOLUTE CARRIER FAMILY 35 protein belonging to a family of proteins involved in catalysing the specific transport of various substrates. Finally, the sequenced fragment containing SNP 27203_199 produced multiple hits hampering the identification of a candidate.

**Table 3:** Three most significant ortho/homologues in *Arabidopsis thaliana* of the five proposed candidates.

Candidate	Name	Score	Similarity	Defline
*Pv5-593.01G186800*	*AT1G66950.1*	2256	72.4	Pleiotropic drug resistance 11
	*AT2G36380.1*	2244	72.7	Pleiotropic drug resistance 6
	*AT1G15520.1*	1825	59.7	Pleiotropic drug resistance 12
*Pv5-593.01G236100*	*AT1G07410.1*	375	85.5	RAB GTPase homolog A2B
	*AT3G46830.1*	351	82.3	RAB GTPase homolog A2C
	*AT1G09630.1*	350	77.2	RAB GTPase 11C
*Pv5-593.05G028500*	*AT1G23780.1*	160	38.4	F-box family protein
	*AT1G23770.1*	129	40.9	F-box family protein
	*AT1G70360.1*	103	36.5	F-box family protein
*Pv5-593.09G089900*	*AT4G31860.1*	602	83.2	Protein phosphatase 2C family protein
	*AT2G25070.2*	577	76.8	Protein phosphatase 2C family protein
	*AT2G25620.2*	148	33.2	DNA-binding protein phosphatase 1
*Pv5-593.11G099400*	*AT5G22090.2*	228	42.2	FAF-like protein (DUF3049)
	*AT5G19260.1*	66	36.6	FANTASTIC four-like protein (DUF3049)
	*AT1G03170.1*	63	36.6	FANTASTIC four-like protein (DUF3049)

### Linkage disequilibrium analysis

**Figure 6:**
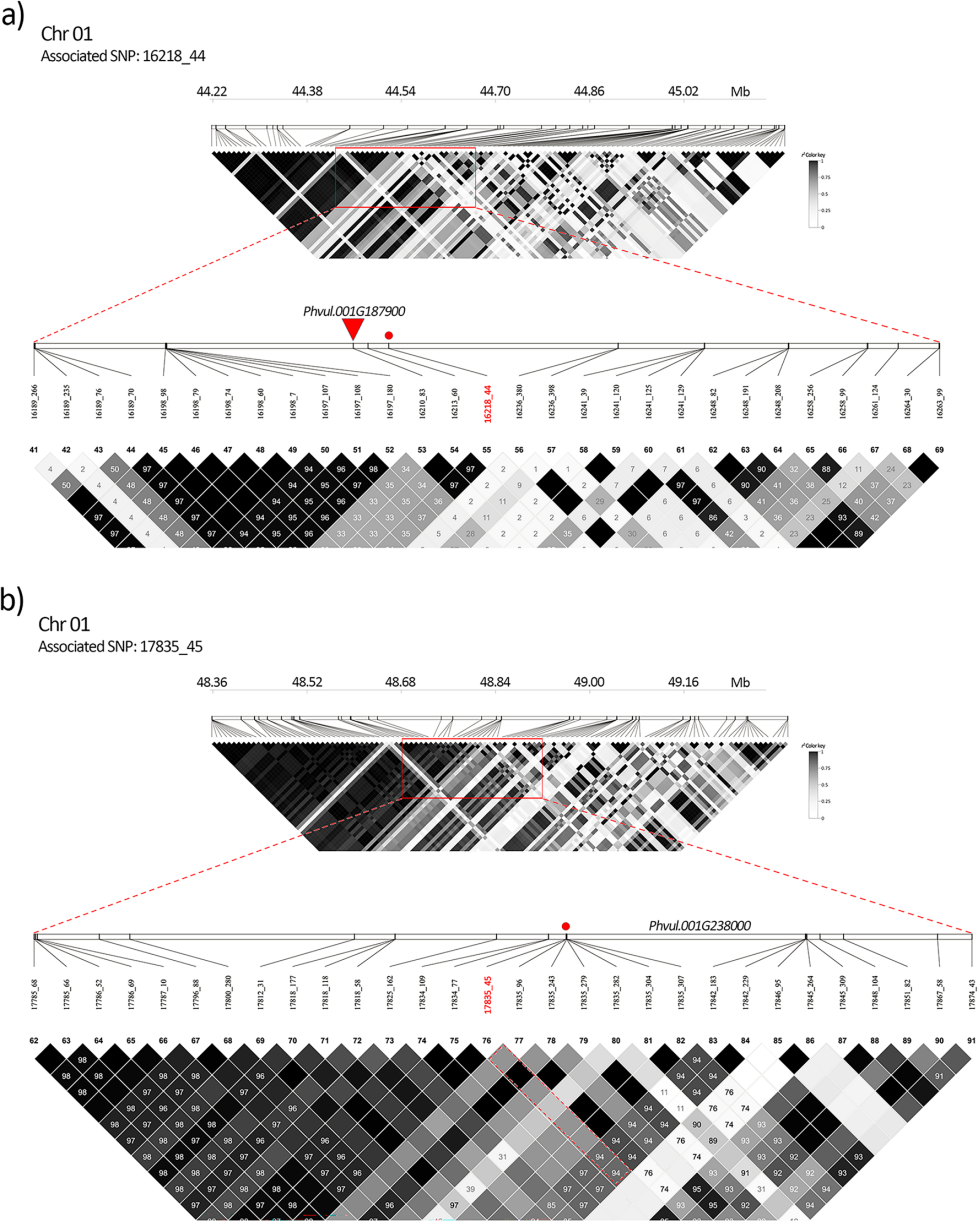
Heatmap (*r*^*2*^) over a ± 0.5 Mb window centred on the significant detected SNP marker (top) and zoom over the chromosomic region containing the associated SNPs and the candidate gene (bottom) of associated SNP 16218_44 (**a**) and 17835_45 (**b**). The name of the significant SNP is highlighted in red; physical position of the SNP (circle) and of the proposed candidate gene (triangle) is also reported. No triangles are showed in (**b**) since none of the displayed SNP is physically located in the candidate gene that is placed between the fragments 17835 and 17842.

According to the results of LD analysis SNP 16218_44 and *Pv5-593.01G186800* belongs to the same recombination block of 3 SNP in high linkage (*r*^*2*^ values from 97 to 100) (Fig. 6a) confirming the almost complete association between the marker and the proposed candidate gene. Similarly, SNP 17835_45 is in strong linkage disequilibrium with the SNP markers flanking the region harbouring the candidate gene *Pv5-593.01G236100*: SNP 17835_96, 17835_243, 17835_279, 17835_282, 17835_304 and 17835_307 before, and SNP 17842_183 and 17842_229 after, respectively (Fig. 6b).

## Discussion

Germination is the first and one of the most important and sensitive stage of the plant life cycle^33^; in legumes, highest sensitivity to salt stress is observed during seedling^34^. According to presented results, the negative impact of salt stress on germination becomes increasingly significant as salinity levels rise, leading to a reduction in the overall percentage of germinated seeds. Such reduction can have detrimental consequences for crop establishment and production^35,36^. Differences between control and salt conditions became evident as early as four days after sowing; germination inhibition continued to increase with higher salinity levels, with the greatest inhibition observed under the highest salt concentration (NaCl 150 mM, Fig. 1). Our findings are consistent and in line with previous studies on common bean^20,23^.

Evidence from the literature suggests that measuring growth inhibition is fast and efficient when quantifying the general effect of salt stress^37^; under adverse conditions, plants allocate resources from standard metabolism and growth to specific stress defence mechanisms to ensure survival^38^. However, this redirection has negative implications for development, particularly for common bean. This aspect being confirmed by the observed aerial and belowground growth parameters significantly impaired by the applied treatment (Fig. 2). Severe reductions of shoot and root growth at similar NaCl concentrations were indeed reported for some common bean cultivars: ICA Pijao^20^ Lody, Gina, Tara^39^ and Tegmen^22^. Similar evidences were also obtained by Kouam et al.^23^ working on eight different common bean genotypes widely grown in Cameroon, and by Çiftçi and colleagues^40^ using 55 bean genotypes collected from Gevaş-Van region in Türkiye. Previous studies have also reported the importance of biomass production under stress and nonstress conditions in common bean; for example Asfaw et al.^41^ showed a moderate phenotypic correlation between shoot biomass and grain yield under both drought stress and nonstress conditions.

Our findings suggest that a level of NaCl 75 mM is highly recommended to screen large diversity panels at seedling stage. Screening at lower salt concentrations could have been less suitable for the identification of tolerant genotypes: in the study by Kouam and colleagues^23^ some of the measured traits (e.g. root length and fresh weight) were not significantly hampered when plants were grown at NaCl 50 mM. According to our results, membership to different genepools do not highlight significant differences in common bean tolerance to salt stress as seedling stage; however, genotypes of Admixed origin in the panel showed a certain degree of tolerance to salt stress with average SDW and RDW values of control vs. stressed not significantly different (Fig. 3). The frequent inter-gene pool hybridization of European common beans^42^ following adaptation to different environmental conditions from “old” to the “new” world could possibly explain this evidence towards salt stress tolerance. Notably, the majority of samples in this diversity panel originate from Southern European landraces^32^ where this species has experienced a secondary diversification, producing new variation^43^.

In recent years, the interest on GWAS arose in both academia and commercial sectors. It is quite direct and relatively straight-forward method to dissect the genetic control of quantitative, complex traits, by screening natural diversity^44^ and sometimes resulting in the identification of genetic variant with relatively high effect, which can be used for breeding purposes. Even if loci for salt tolerance have been identified in other grain crops, like rice, barley, soybean, wheat and field pea^45^, to the best of our knowledge, this is the first study where GWAS has led to the identification of genes involved in salinity tolerance in common bean. Already successfully used to perform GWAS for flowering related traits^31^ and zinc seed content^46^, the characterisation of the diversity panel developed at the University of Perugia allowed the identification of seven SNP significantly associated with salt stress tolerance (Fig. 4) and meaningful candidate genes are proposed for five SNP.

Phosphatase are known to play a critical role in regulating abiotic stress tolerance in plants being involved in stress signaling which mediates activation of stress tolerance^47^. Remarkably, in Molina et al.^48^ a gene annotated as “protein phosphatase 2C-like protein (*AT4g31860/F11C18*)”, the Arabidopsis gene most similar to our candidate *Pv5-593.09G089900* (Table 3), was listed among genes responsive to drought stress in the chickpea roots transcriptome while Roy and colleague^49^ showed that in Arabidopsis, the gene *AT2g25070*, which is the second most similar Arabidopsis gene to our candidate (Table 3), is located within a highly significant QTL linked to Na^+^ exclusion identified through successive rounds of fine mapping^50^.

Although a precise function for *FAF* genes has yet to be sufficiently determined, they can be functionally associated with an abiotic stress response. For example, in *Capsicum annum, CaFAF1*-silenced plants exhibited enhanced drought stress tolerance and enhanced abscisic acid-mediated stomatal closure^51^ in comparison to controls. Indeed, different studies have shown that drought resistance is correlated with the expression of stress-related marker genes^52,53^ suggesting a possible role for the gene in salt stress response. According to Wang et al.^54^ the Arabidopsis homolog of our candidate *Pv5-593.11G099400* (*AT5G22090, EAR1*) encodes a protein that interacts with the N-terminal inhibition domains of all the six 2C-type protein phosphatases (PP2Cs) during ABA signaling and enhances the activity of PP2Cs. Interesting enough, testing drought tolerance, the authors observed that the rate of water loss was much slower in *EAR1* mutants than in the wild type at various time points.

*Pv5-593.01G186800* encodes for an ABC transporter-like protein; ABC transporters are abundant in the genomes of both prokaryotes and eukaryotes^55^. Plants are particularly rich in ABC proteins, many of them behave as ATP-dependent cassette transporters (coupled with ATP hydrolysis) playing active role in the transport of wide range of substrates across biological membranes; they are involved in different process including, lipid catabolism, xenobiotic detoxification^56^, disease resistance, stomatal function, and biotic and abiotic stresses tolerance^57^. Several reports state that in response to salt stress, ABC transporters showed differential expression and played key roles in developing salt tolerance in plants^58^. This class of genes has been identified among those differentially expressed in the analysis of root and leaf transcriptomes of a salt tolerant common bean genotype grown under salt stress and control conditions^59^. In a phylogenetic and expression analysis of ATP-binding cassette transporters in *Oryza sativa* Saha and colleagues^60^ showed that in RILs with different salt tolerance, the expression level of members of this gene family is modulated by salt stress (both up or down regulated). Another member (36) of the ABC transporter G family in Arabidopsis promotes resistance to abiotic stresses (e.g. drought and salt stress) and favours general growth by preventing sodium accumulation in plants^61^. Transgenic Arabidopsis plants overexpressing ABCG36 exhibits a much higher Fresh Weight (FW) than wild-type plants when exposed to drought stress^61^. RABA2B Ras-related proteins are also mainly involved in the vesicular trafficking machinery^62^ that plays an important role in the mediation of plant responses to a range of abiotic stresses^63^; according to several studies these genes are often highly expressed in response to biotic and abiotic stresses^64^. Interestingly enough, Rab GTPase binding is among the GO terms identified in a GO enrichment analysis of salt-related genes during the sprout stage of common bean under salt stress conditions^65^.In a recent work in Arabidopsis, Ambastha et al.^66^ showed that RabA2b, the best hit for this species of our candidate *Pv5-593.01G236100* (Table 3), is highly upregulated by several abiotic stresses including drought and salt being the promoter activity induced by osmotic stresses—while the rest of the RabA2 members generally responded marginally to these stresses—and that transgenic plants overexpressing *RabA2b* are “strikingly drought resistant” due to an enrichment in the plasma membrane of stress-coping proteins as well as of cell wall/cuticle modifiers; cuticle permeability of transgenic leaves was also significantly reduced.

Finally, *Pv5-593.05G028500*, directly associated with SNP 70878_186, codes for a F-box protein 7 (FBXO7). In plants F-box genes form one of the largest multigene superfamilies and control many important biological functions including response to abiotic stresses^67,68^. In Rao et al.^69^ At1g23780, the Arabidopsis gene most similar to our candidate (Table 3), is listed among proteins specifically interacting with SKP1-like protein13 (ASK13); ASK13 is differentially regulated in different organs during seed development and germination and is up-regulated in response to abiotic stress. Interestingly, enough At1g23780 is also predominantly expressed in seeds. According to the authors of the study, the similar expression patterns between the F-box protein and ASK13 further adds to the possibility of several potential ASK13–E3s that may be implicated in regulating seed germination and seedling growth under abiotic stress conditions, possibly through modulating ROS accumulation^69^. Even more striking, according to Vlad and colleagues^70^, At1g23780 is among the ‘top 10’ putative CDPK–SnRK kinase substrates found in Arabidopsis; in particular it is part of the group of “SOS2 (for SALT OVERLY SENSITIVE2) putative substrates in the Arabidopsis protein database”. SOS2, and its protein-interacting partner (SOS3), have been described as central players in salt-stress responses in Arabidopsis where loss of SOS2, SOS3, and SOS1 proteins results in different levels of NaCl hypersensitivity^71^.

Evidence from the literature confirm the value of the proposed candidates in playing a role in common bean’s response to salt or drought stress. Indeed, it has been established that the mechanisms underlying the plant responses to drought and salt stress are broadly comparable^72^. In certain instances, mutations in orthologs/homologs of our candidates have been observed to enhance drought or salt tolerance in other species, additionally, some of these genes are found within QTLs associated with mechanisms utilized by plants to withstand salt stress (e.g. salt exclusion). Certain candidates are members of gene families whose expression is influenced by salt stress, providing significant evidence for their role in regulating salt tolerance.

## Conclusions

By leveraging the natural variation in salt stress tolerance among the accessions in the diversity panel conserved in the Department Gene Bank (FAO code: ITA 363) we were able to get some first insights on the genetic architecture underlying salt tolerance traits at the seedling stage in common bean. Our finding provide support for the possible role of the proposed candidate genes, as their functions have been validated in homologous genes in other species. Due to the limited understanding of the genetic control of salt stress tolerance in common bean, it is challenging to validate the candidates by comparing their positions with known QTLs or genes already known to be involved in the process. However, the fairly good indications of the potential role of the candidate genes identified in this research in increasing salt stress tolerance are worth further research efforts. In this regard, it is worth noting that the diversity panel used in this study has been already successfully used to identify genes involved in flowering time control as well as in zinc content in seeds. The here reported evidence improves the understanding of the molecular mechanisms and pathways regulating seedling salt tolerance in common bean that include, among others, vesicular trafficking machinery, transport across biological membranes as well as post-translational modifications, and provides valuable insights that can be utilized by breeders to improve the salt stress tolerance of common beans. Identifying genes responsible for enhanced salt tolerance in beans is important for genetic improvement programs aimed at developing new, more resilient varieties that can also cope with salt stress; such materials are urgently needed due to population growth and the challenges posed by climate change.

## Materials and methods

Acquisition of all plant materials and all methods were performed in accordance with the relevant guidelines and regulations.

### The collection

The diversity panel of 192 common bean genotypes (NCBI accession codes from SAMN12035168 to SAMN12035359) developed by the Department of Agricultural, Food and Environmental Sciences (DSA3) of the University of Perugia was used as starting material for this study. This panel was developed using Single Seed Descent (SSD) for 5 consecutive generations under isolation, resulting in the production of highly homozygous lines (hereafter called genotypes)^31,32^. A previous genetic characterization of these genotypes, based on a double digest Restriction-site Associated DNA sequencing (ddRAD-seq), allowed to depict both population structure and genotype cryptic relatedness (kinship) as detailed in^31,32^. Results of the Evanno test clearly indicated K = 2 as the most suitable level of population subdivision to explain the genetic structure of the studied panel^31^ (Fig. S2, Supplementary Materials): cluster 1 (K1) holds 87 genotypes of Mesoamerican origin, cluster 2 (K2) 94 genotypes of Andean origin while 11 genotypes are admixed considering a threshold of *q* ≥ 0.8^73,74^. The kinship analysis showed different relatedness levels among genotypes and highlighted no genetic redundancy^31^.

### Setting up salt stress experimental conditions

#### Salt tolerance assessment at germination

Six genotypes were selected for a seed germination experiment under varying salt stress conditions (Table S3, Supplementary Materials). Prior to germination, the seeds were surface-sterilized using the protocol outlined by Parsa and colleagues with slight modifications^75^ as follows: (1) wash: Triton X-100 (Sigma, St. Louis, MO, USA) 0.1%, 2 min; (2) sterilization A: 0.5% sodium hypochlorite, 2 min; (3) sterilization B: 70% ethyl alcohol, 2 min and (4) rinsing: double distilled water, for 20 s. Samples were dried with filter paper, placed in vacuum sealed aluminium bags and stored at 5 °C. The germination test was conducted on sterilized quartz sand substrate in Petri dishes using, employing four different treatments: 15 ml distilled water at different NaCl solutions: 0, 50, 100 and 150 mM. For each of the tested genotypes (n = 6) and treatments(n = 4) we assesed three biological replicates with each replica consisting of 12 seeds, arranged in 2 Petri dishes; 144 seeds per genotype (12 seeds × 4 treatments × 3 replicates) were tested for a grand total of 864 tested individual seeds. Germination ability was recorded following the International Seed Testing Agency procedures^76^.

Number of germinated seeds was recorded daily from the third to the seventh day after the beginning of the test. Germination process was described with the following indexes: *Germination Rate* (GR), *Germination Potential* (GP) and *Germination Index* (GI) as in^77^. . GR = (*G7*/*TS*) × 100; GP = (*G3*/*TS*) × 100 where *TS* is the is the total number of seeds and *G3* or *G7* is the number of germinated seeds from the first to the third (*G3*) or seventh (*G7*) day after sowing;

$$\mathrm{and GI}={\sum }_{1\to n}^{t}\frac{Gt}{Dt},$$

where *t* is the number of days after planting, *Gt* is the number of germinated seeds at the *t*th day after sowing, *Dt* is the number of days after the beginning, corresponding to *Gt*. Different descriptive statistics were computed on the calculated indexes, significance of pair-wise differences—between control and the different applied treatments—was assessed by Student's t-test; before applying the test, GR and GP were subjected to angular transformation (2 × arcsine of square root of the proportion).

#### Salt tolerance assessment on seedlings

At the conclusion of the germination test, seedlings successfully germinated under NaCl 0 mM (control), 100 mM and 150 mM were transferred in hydroponic prepared using Hoagland's nutrient solution with the corresponding NaCl concentrations^78^. Twelve germinated seeds of four (out of six) genotypes were transferred from Petri dishes to floating cylindrical sponges and grown for 11 days at the three conditions (NaCl 0, 100 and 150 mM), in a controlled environment: temperature of 20 °C (± 2 °C) under pairs of LED tubes (36 W, 120 × 26 mm) with a 160° beam angle and a luminous flux of 860 lumens (lm) for 12 h per day (Fig. S4, Supplementary Materials). Different stem and root traits were recorded on for each genotype as in^77^; traits are listed in Table 4. Data were processed by ANOVA using a linear model where the individual value Y_ijk_ of the levels i of the fixed effect “Entry” (E), j of the fixed effect “Treatment” (T) and k of the fixed effect “Block” (B) is:

$${\text{Y}}_{{{\text{ijk}}}} = {\text{ m }} + {\text{ E}}_{{\text{i}}} + {\text{ T}}_{{\text{j}}} + {\text{ B}}_{{\text{k}}} + {\text{ (ET)}}_{{{\text{ij}}}} + {\text{ (EB)}}_{{{\text{ik}}}} + {\text{ (TB)}}_{{{\text{jk}}}} + {\text{ (ETB)}}_{{{\text{ijk}}}} + {\text{ e}}_{{{\text{ijk}}}} .$$

where m is the grand mean and eijkz is the experimental error.

**Table 4:** List of morphological traits recorded on plants grown in hydroponic culture.

Trait	Acronym	Measure unit
Root length	RL	cm
Root fresh weight	RFW	g
Root dry weight	RDW	g
Shoot length	SL	cm
Shoot fresh weight	SFW	g
Shoot dry weight	SDW	g

### Characterisation of the common bean collection

#### Phenotypic characterisation

Following the initial test, we expanded the salt stress tolerance experiment to include the whole common bean diversity panel. A concentration 75 mM NaCl was determined as optimal salt stress level for this large-scale screening. For each genotype, 12 seeds were sterilized using the same protocol described above. Seeds were equally split into two Petri dishes, under control (0 mM NaCl) and salt stress (75 mM NaCl) treatment, respectively. After 7 days, seedlings were transferred into hydroponic culture, keeping the same NaCl conditions. The hydroponic culture solutions and growing conditions remained consistent with the previously described methods. For each genotype, 3 biological replicates were grown for each treatment. After 10 days root (RDW) and shoot dry weight (SDW) were measured. Unless specified otherwise, the following data analyses were performed in R^79^. Descriptive statistics and best linear unbiased predictors (BLUPs) were estimated with *r/agricolae*^80^ and results plotted with *r/ggplot2*^81^. Effects of geographical origin, phaseolin type, growth habit as well as genome-wide ancestry membership (Table 5)^31,32^ was tested to explain observed variation of on shoot and root development under control and stress conditions.

**Table 5:** Factors tested for possible effect on plant development under control and salt-stress conditions.

Factor name	Description	Possible Values
Continent	Continent of origin of the original landrace	America South, America Center and Europe
Phaseolin	Type of phaseolin	C, T and S
Structure	Main STRUCTURE group assignation	K1^a^, K2^b^, and Admixed (ADM)^c^
Growth habit	Type of growth of the plant	Prostrate-indeterminate, Bush-indeterminate, Bush and Climbing

^a^Mainly including accessions of Mesoamerican origin.

^b^Mainly including accessions of Andean origin.

^c^Including all genotypes with a STRUCTURE assignment value *q* ≤ 0.8.

Variance components were also used to estimate broad-sense heritability (*He*^2^_*B*_) as follows:

$${He}_{B}^{2}=\frac{{\sigma }_{g}^{2}}{{\sigma }_{p}^{2}}\times 100$$

Genotype Salt Stress Tolerance ability was described by the reciprocal, normalized difference between shoot and root dry weight under controlled and stressed conditions, respectively. This value was expressed as Salt Stress Tolerance Coefficient (SSTC) as follows:

$$D{W}_{SSTC}=1-\frac{D{W}_{control}-D{W}_{stress}}{D{W}_{control}}$$

#### Genotypic characterisation

Genetic diversity of the panel was characterised by means of double digest Restriction-site Associated DNA sequencing (ddRAD-seq) using *SphI* and *MboI* on Illumina HiSeq2500 platform for sequencing (Illumina, San Diego, California, USA) as previously described by Caproni et al.^32^ and Raggi et al.^82^. Demultiplexing of raw Illumina sequences was performed using Stacks v 2.0^83^ and subsequent alignment to the common bean reference genome using BWA-MEM^84^ with default parameters. Stacks v2.0 was also used to detect all the covered SNP loci from the aligned reads (minimum cover depth 6x) and to filter the detected loci using the population program (included in Stacks v2.0). In this last step, only loci that are represented in at least 75% of the population were retained.

Loci and genotypes with a missingness rate ≥ 10%, a minor allele frequency (MAF) ≤ 5% and a heterozygosity ≥ 2% were removed. The final dataset consisted of 49,518 SNPs.

To further explore the genetic structure of the panel, a set of 2518 SNPs, which were pruned for linkage disequilibrium (r^2^ < 0.3) based on previous work by Raggi et al.^82^, was extracted. Principal Component Analysis (PCA) was performed on this LD-pruned SNP dataset using *r/adegenet*^85^.

#### Genome wide association analysis

Associations of salt tolerance indexes of root (RDW_SSTC) and shoot (SDW_SSTC) were tested via GWAS. The analysis was performed using a Fixed and Random model Circulating Probability Unification (FarmCPU)^86^ as implemented in *r/MVP*^87^. considering the first 10 genetic PCs and the kinship as covariates, both estimated using the LD-pruned set. The best number of PCs to be retained in the analysis was determined using a procedure adapted from the study of Woldeyohannes et al.^88^. Briefly, the FarmCPU regression was applied with different number of PCs starting from 5. Following the first round of mapping, individual QQ plots were visually examined to identify any inflation in the distribution of p-values result. Upon detecting inflation, the corresponding GWAS scan was rerun, incorporating an additional 5 genetic PCs as covariates, until optimal model fit was observed. Kinship was calculated using the method from VanRaden^89^.

The GWAS scans were run with the full set of markers (49,518) while Bonferroni multiple testing threshold was set on SNPs in Linkage Equilibrium (i.e. LD-pruned set) and an alpha = 0.01. For markers significantly associated with target phenotypes and physically located within genes, corresponding genes were identified using the BLAST tool in Phytozome 13^90^ against the *Phaseolus vulgaris 5-593 v1.1* genome. In the other cases (i.e. significant SNPs not located within genes), relevant candidate genes where detected by proximity, using the “Jbrowse” tool^91^ and using a window of maximum ± 50 kb^31,92^, and by gene functional annotation also using the aminoacidic sequences of the putative candidates as query against the *Arabidopsis thaliana* protein database (Araport11 protein sequences) using the online tool BLASTP (AA query, AA db).

#### Linkage disequilibrium analysis

To ascertain whether the identified SNP markers located in non-coding regions and candidate genes are in Linkage Disequilibrium (LD)—meaning that they tend to be inherited together—a LD analysis was carried out in HaploView 4.288. Pairwise LD between markers (*r*^*2*^) was calculated within a window of ± 0.5 Mb around the most significant marker associated with the corresponding trait. To better visualise LD patterns between a candidate gene and the associated markers, further analyses were performed and visualised in narrower windows.

### Supplementary Information

Supplementary Figures.

Supplementary Tables.

## Data Availability

Descriptive information, including metadata, about the biological materials analysed in this study are available in the National Center for Biotechnology Information (NCBI) BioSample database from ID SAMN12035168 to SAMN12035359. Raw DNA ddRAD sequencing reads are available at the European Nucleotide archive under the ID PRJEB33063 (https://www.ebi.ac.uk/ena/data/view/). Raw phenotyping data are available in Tables S4 and S5 (Supplementary Materials) for shoot and root, respectively. In the cited databases, genotypes are named consistently.
