# Supplementary material for: Investigating the genetic basis of salt-tolerance in common bean: a genome-wide association study at the early vegetative stage
Source: Sci Rep. 2024 Mar 4;14:5315. doi: 10.1038/s41598-024-55403-z (PMC10912697; doi:10.1038/s41598-024-55403-z)
Supplement: Supplementary file 1 — Supplementary Figures. [file 41598_2024_55403_MOESM1_ESM.docx]

**Investigating the Genetic Basis of Salt-Tolerance in Common Bean: a Genome-Wide Association Study at the Early Vegetative Stage**

**Lorenzo Raggi^1*^, Leonardo Caproni^1,2^, Simona Ciancaleoni^1^, Roberto D’Amato^1^, Daniela Businelli^1^ and Valeria Negri^1^**

^1^ Dipartimento di Scienze Agrarie Alimentari e Ambientali (DSA3), Università degli Studi di Perugia, Perugia, Italy

^2^ Present affiliation: Center of Plant Sciences, Scuola Superiore Sant’Anna, Pisa, Italy

* lorenzo.raggi@unipg.it


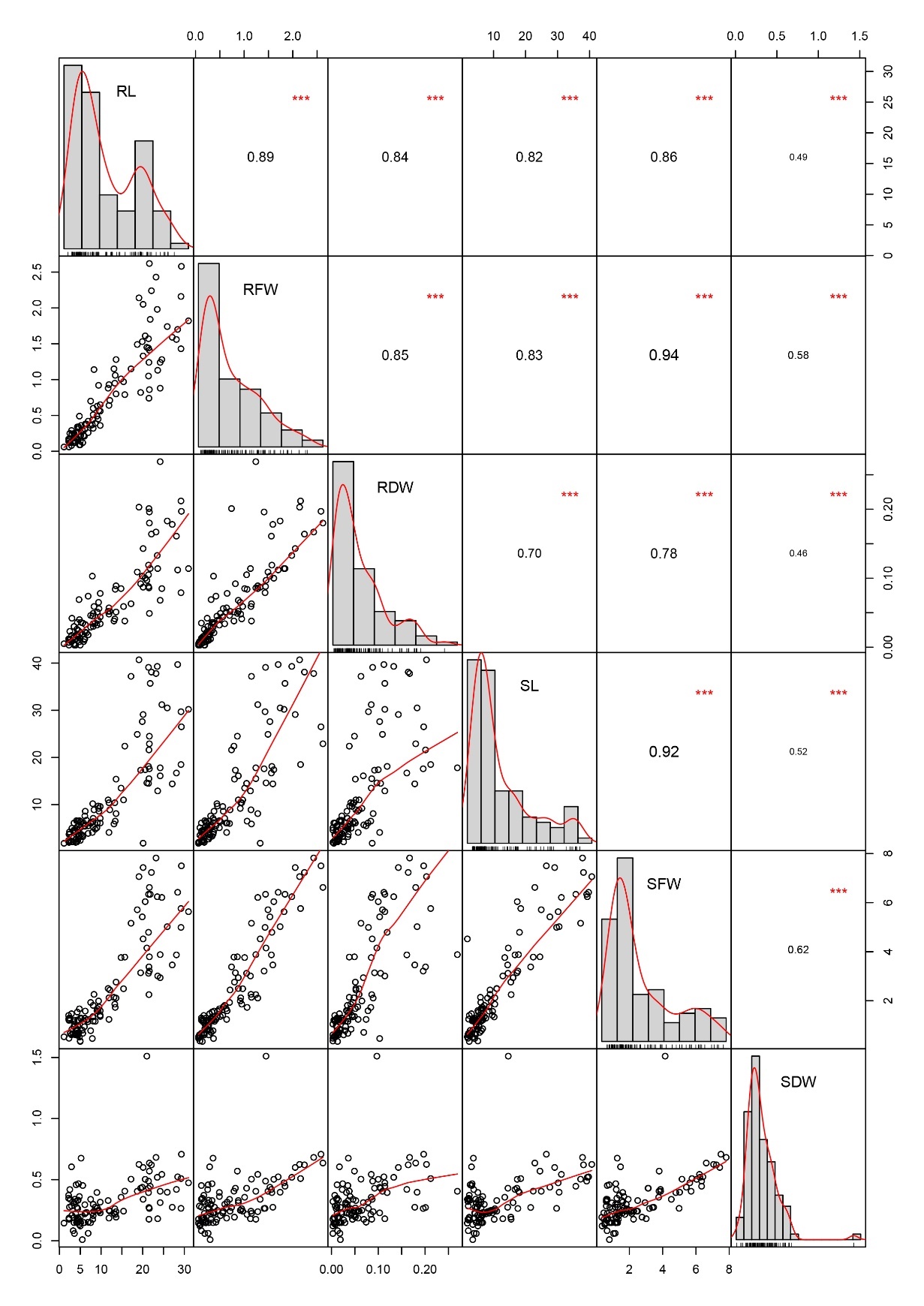


**Figure S1.** Pearson correlation coefficient among recorded traits with corresponding significance of detected correlation (above diagonal), x y scatter plots of recorded data (below diagonal) and histograms of distribution (diagonal). RL=root length, RFW=root fresh weight, RDW=root dry weight, SL=shoot length, SFW=shoot fresh weight and SDW=shoot dry weight.


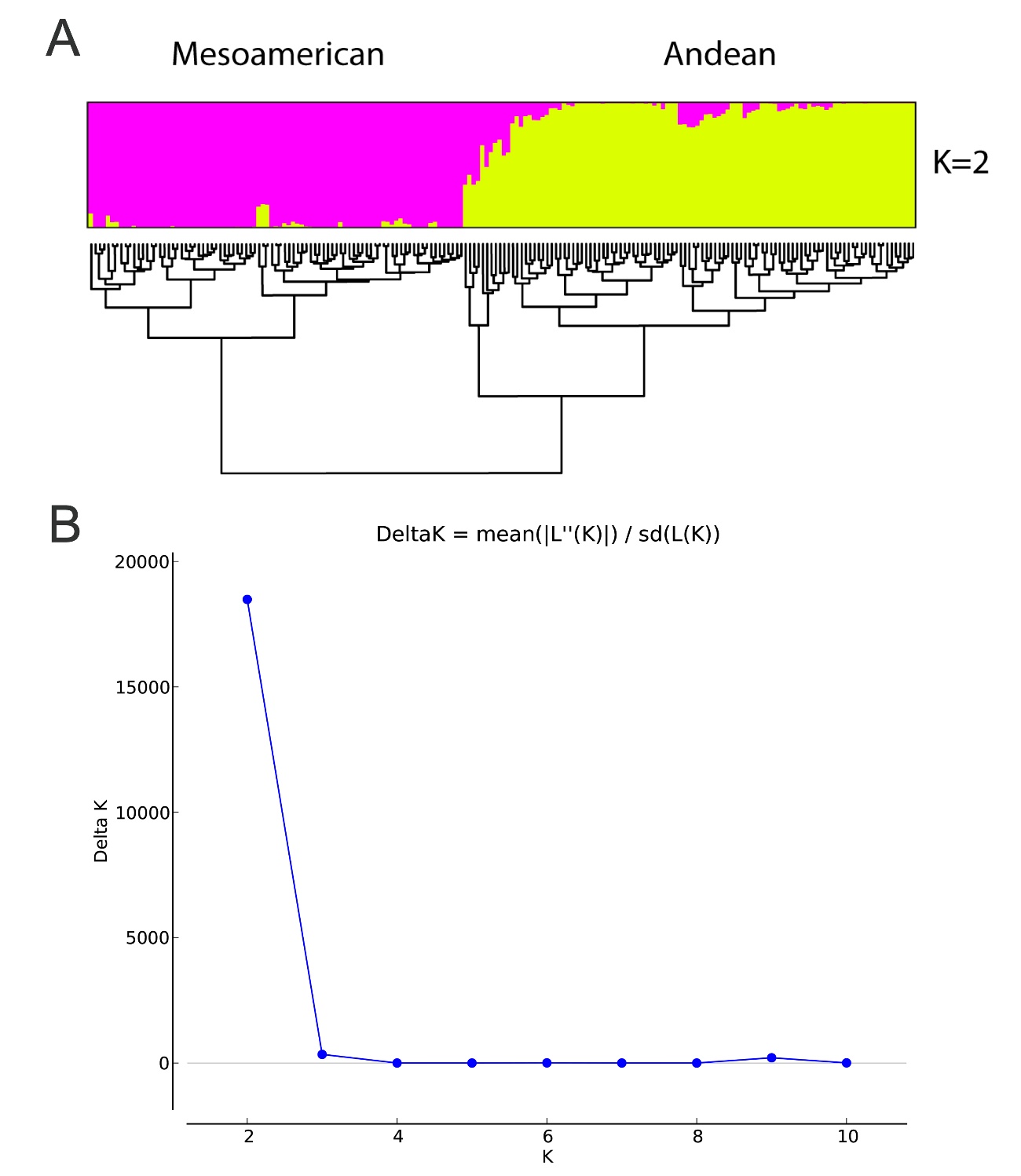


**Figure S2.** Genetic structure of the studied collection at K2 (A). Results of the Evanno test indicating K = 2 as the most suitable level of population subdivision (B). Adapted from Caproni et al. (2019) doi:10.3390/su11195443


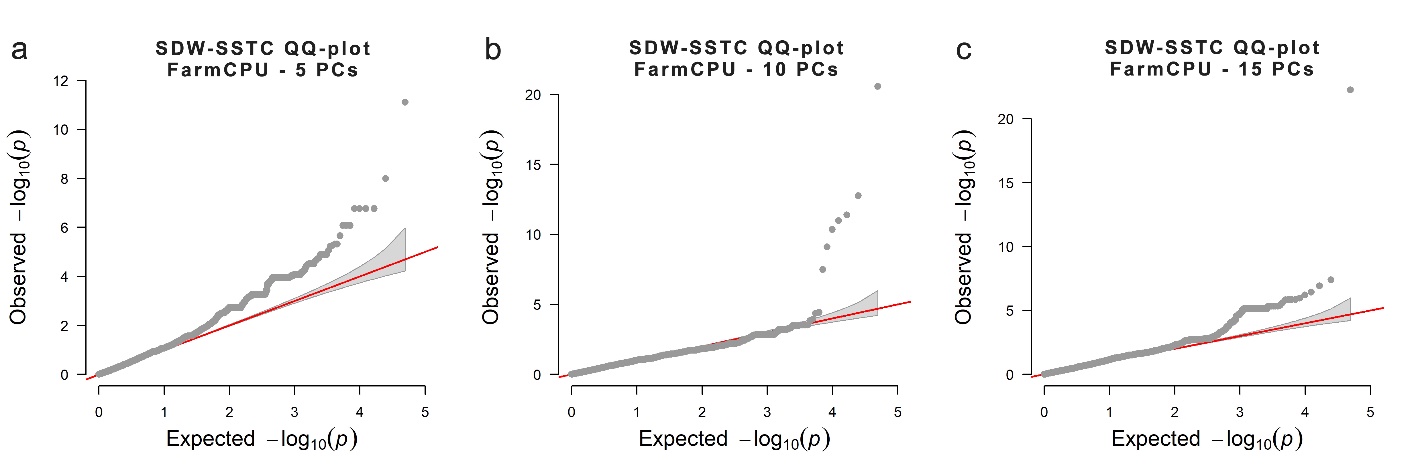


**Figure S3.** Individual QQ plots obtained retaining 5 (a), 10 (b) PCs for the trait Shoot Dry Weight Salt Stress Tolerance Coefficient.


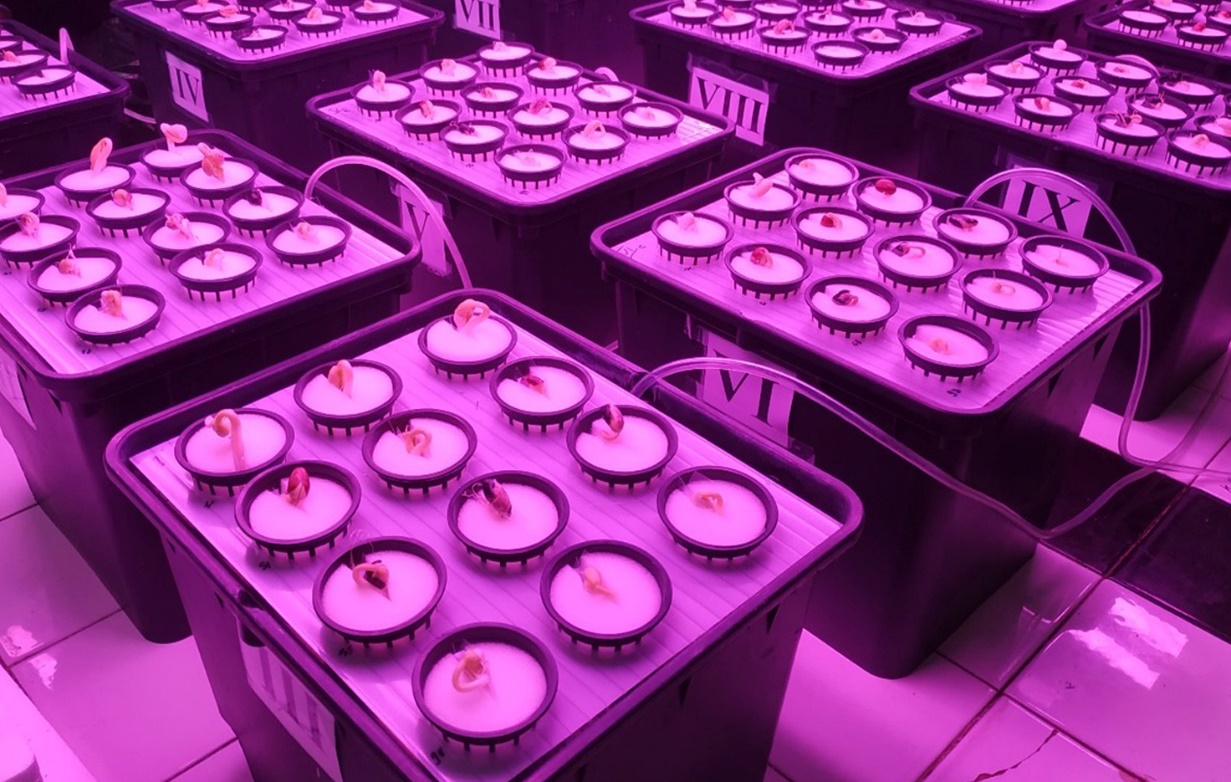


**Figure S4.** Common bean seedlings grown hydroponically.
